# Identification of nuclear export inhibitor-based combination therapies in preclinical models of triple-negative breast cancer

**DOI:** 10.1016/j.tranon.2021.101235

**Published:** 2021-10-07

**Authors:** Narmeen S. Rashid, Nicole S. Hairr, Graeme Murray, Amy L. Olex, Tess J. Leftwich, Jacqueline M. Grible, Jason Reed, Mikhail G. Dozmorov, J. Chuck Harrell

**Affiliations:** a Department of Pathology, School of Medicine, Virginia Commonwealth University, 1101 East Marshall St, Office 4-007, P.O. Box 980662, Richmond, VA 23298-0662, USA; b Department of Biology, University of Richmond, Richmond, VA USA; c C. Kenneth and Diane Wright Center for Clinical and Translational Research, Virginia Commonwealth University, Richmond, VA USA; d Massey Cancer Center, Virginia Commonwealth University, Richmond, VA USA; e Department of Physics, Virginia Commonwealth University, Richmond, VA USA; f Department of Biostatistics, Virginia Commonwealth University, Richmond, VA USA

**Keywords:** Breast cancer, TNBC, KPT-330, GSK2126458, XPO1

## Abstract

An estimated 284,000 Americans will be diagnosed with breast cancer in 2021. Of these individuals, 15–20% have basal-like triple-negative breast cancer (TNBC), which is known to be highly metastatic. Chemotherapy is standard of care for TNBC patients, but chemoresistance is a common clinical problem. There is currently a lack of alternative, targeted treatment strategies for TNBC; this study sought to identify novel therapeutic combinations to treat basal-like TNBCs. For these studies, four human basal-like TNBC cell lines were utilized to determine the cytotoxicity profile of 1363 clinically-used drugs. Ten promising therapeutic candidates were identified, and synergism studies were performed *in vitro*. Two drug combinations that included KPT-330, an XPO1 inhibitor, were synergistic in all four cell lines. *In vivo* testing of four basal-like patient-derived xenografts (PDX) identified one combination, KPT-330 and GSK2126458 (a PI3K/mTOR inhibitor), that decreased tumor burden in mice significantly more than monotherapy with either single agent. Bulk and single-cell RNA-sequencing, immunohistochemistry, and analysis of published genomic datasets found that XPO1 was abundantly expressed in human basal-like TNBC cell lines, PDXs, and patient tumor samples. Within basal-like PDXs, XPO1 overexpression was associated with increased proliferation at the cellular level. Within patient datasets, XPO1 overexpression was correlated with greater rates of metastasis in patients with basal-like tumors. These studies identify a promising potential new combination therapy for patients with basal-like breast cancer.

## Highlights

• High-throughput drug screening reveals promising therapeutic candidates for TNBC.
• KPT-330, an XPO1 inhibitor, and GSK2126458 exhibit synergism in preclinical models of TNBC.
• XPO1 is overexpressed in basal-like breast tumors.
• XPO1 expression is associated with PIK3CA, MTOR, and MKI67 expression at the single-cell level.
• XPO1 overexpression in basal-like patients is associated with greater rates of metastases.

## Introduction

Breast cancer is the most frequently diagnosed cancer in women, contributing 23% of total cancer diagnoses and 14% of total cancer deaths [1]. It is estimated that over 284,000 Americans will be diagnosed with breast cancer in 2021 [2]. Of these individuals, approximately 10–20% will be diagnosed with triple-negative breast cancer (TNBC) [3]. TNBC is characterized by a lack of estrogen receptor (ER), progesterone receptor (PR), and human epidermal growth factor receptor 2 (HER2) amplification [4]. TNBC is an aggressive, highly metastatic subtype of breast cancer [5]. Despite greater initial clinical response to neoadjuvant chemotherapy, patients with TNBC have a higher likelihood of distant reoccurrence and a lower rate of survival than patients with other breast cancer subtypes [6,7]. TNBC is difficult to treat due to its heterogeneity and lack of established biomarkers. [8] Gene expression profiles reveal at least six distinct TNBC subtypes: basal-like 1 and 2, immunomodulatory, mesenchymal, mesenchymal stem-like, and luminal androgen receptor [8]. The majority of TNBCs are basal-like [9].

Unlike ER+, PR+, or HER2+ breast cancers, TNBCs cannot be treated with endocrine therapies or HER2-targeted agents, and chemotherapies are standard of care. Platinum-based compounds carboplatin and cisplatin are often first-line therapies for basal-like TNBC [10]. Second line therapies include combination treatment with multiple chemotherapeutics from different classes [10]. Unfortunately, drug resistance can occur, and 90% of treatment failures in metastatic cancers are attributed to chemoresistance [11]. Tumor environmental stresses induced by chemotherapies can promote autophagy and senescence, both of which contribute to the development of chemoresistance in TNBC [12]. Given that many basal-like tumors are intrinsically chemotherapy resistant, or develop acquired resistance, it is important to identify targeted therapeutics that can be incorporated into the standard of care. With thousands of available drugs, high throughput drug screening (HTS) is a systematic method of identifying promising therapeutic agents. Previously, HTS has been used to identify an antineoplastic agent, A-105972, that showed promising *in vivo* activity, increasing the life span of mice with melanoma and leukemia [13]. Powell et al. (2020) performed HTS on treatment-naïve TNBC samples to identify promising drugs that are cytotoxic towards different TNBC subtypes [14]. HTS has also been used to identify synergistic drug combinations [[15], [16], [17]].

Due to high rates of metastasis, basal-like disease is associated with relatively worse prognosis than other TNBC subtypes [18,19] The present study focuses on identifying targeted drug pairs that are cytotoxic to basal-like TNBC. Through screening a drug library containing 1363 drugs-most of which are FDA-approved- on basal-like cell lines and performing synergism studies on selected combinations, synergistically cytotoxic therapeutic combinations were identified. Then, the antitumor efficacy of these combinations was assessed *in vivo* using human basal-like TNBC PDXs. Single-cell RNA sequencing (scRNAseq) and immunohistochemistry assessed the expression of drug targets in human basal-like TNBC cell lines and PDXs. As presented here, these pre-clinical studies suggest that co-targeting XPO1 and PI3K/mTOR is a promising approach for the treatment of basal-like breast cancer.

## Material and methods

### Breast cancer cell lines

Four basal-like TNBC cell lines- MDA-468, HCC-1143, HCC-1187, and SUM-149- were used in this study. MDA-468 cells were provided by Dr. Youngman Oh (VCU). SUM-149 cells were purchased from Asterand. HCC-1143 and HCC-1187 cells were purchased from the American Type Culture Collection (ATCC). Cells were cultured in RPMI-1640 GlutaMAX media (ThermoFisher Scientific) supplemented with 10% fetal bovine serum (FBS) and penicillin/streptomycin. Cells were negative for mycoplasma infection (ATCC Mycoplasma Detection Kit).

### Cell viability assays

Firefly luciferase-GFP lentiviral transduction was performed on all cell lines to induce luciferase expression. CMV-Luciferase (firefly)−2A-GFP was purchased from GenTarget Inc. Puromycin was used to select for labeled cells to establish stable, GFP-luciferase labeled cell lines. Cell lines were plated in 96-well plates at 1500 to 5000 cells (cell-line dependent) per well. Cells were incubated overnight to allow for adherence. Then, cells were treated with drugs for 72 h. At hour 72, cells were imaged to measure luciferase activity (total photon flux per second) two minutes after the addition of d-luciferin (15 mg/ml; GoldBio) (1/20 of total volume per well). The IVIS Spectrum *In vivo* Imaging System (Xenogen IVIS-200) and living image software (PerkinElmer) were used to image cells and quantify luciferase activity [17]. Labeled cell lines and luciferase readout were used for high-throughput drug screening and single-dose combination assay. Unlabeled, parental cell lines and CellTiter-Glo Luminescent Viability Assay (Promega) were used for synergism screens. CellTiter-Glo Luminescent Viability Assay was performed according to the manufacturer's protocol.

### *In vitro* high throughput drug screening

MDA-468, HCC-1143, HCC-1187, and SUM-149 cells were treated with 1363 drugs (ApexBio DiscoveryProbe FDA-approved Drug Library) at 10 µM for 72 h. Cell viability was quantified by normalizing treated wells to vehicle wells to produce a percent of vehicle value. The 1363 drugs were ranked by cytotoxicity in each cell line. Venny (https://bioinfogp.cnb.csic.es/tools/venny/) was used for cytotoxicity visualization. 64 of these drugs displayed exceptional relative cytotoxicity across all four cell lines. From these 64 drugs, six drugs targeting different genes were selected for further study: MLN2238, crizotinib, afatinib, KPT-330, dovitinib, and GSK2126458. ABT-263 and dasatinib were also included, as they showed exceptional cytotoxicity in three of the four cell lines. Finally, sorafenib and cobimetinib were selected due to their unique gene targets, BRAF and MEK respectively. Sorafenib and cobimetinib were the most cytotoxic BRAF and MEK inhibitors studied. Heatmap depicting relative cytotoxicity of 68 drugs of interest on human basal-like TNBC cell lines and PDXs was created using Morpheus (https://software.broadinstitute.org/morpheus). Data were hierarchically clustered by both samples (cell lines/PDXs) and drugs using the one minus Pearson correlation metric and average linkage method.

### Single-dose two-drug combination studies

The cell lines HCC-1143 and SUM-149 were used to perform all preliminary combination studies. Dose response curves were performed to identify the cytotoxicity profiles of the ten drugs of interest. Solid drug was purchased from ApexBio. Dose response curves were used to identify a cytotoxic dose, or approximate IC50 dose, of each drug. Then, cells were treated with the identified dose of each drug alone and in combination with the identified dose of all nine other drugs. The cytotoxicity of each combination was rank ordered to identify the most cytotoxic combinations in each cell line. The cytotoxicity of the combination was compared to the cytotoxicity of both drugs alone. In order to be selected for further study, the cytotoxicity of the combination had to be greater than the cytotoxicity of either single agent. Six combinations that met these criteria in HCC-1143 and/or SUM-149 were selected for further study.

### Synergism studies

Unlabeled human basal-like TNBC cell lines were treated with seven doses of each drug in the combination and all two-drug dose combinations for 72 h *in vitro*. Two independent experiments were performed in triplicate. The data were analyzed with CompuSyn software, which utilizes the Chou-Talalay method to identify quantifiable synergism between two or more drugs [20], [21], [22]. CompuSyn software produced CI values and DRI values for each independent experiment. CI values and DRI values were averaged for each cell line to produce Fa-CI and Fa-DRI plots.

### High speed live cell interferometry

UCD52 mammary gland tumors were excised from mice once they reached ∼10 mm x 10 mm in size. Tumors were prepped into a single-cell suspension using the protocol described previously [17]. Cells were plated in a 24-well plate at a density of 10^3^–10^4^ cells per well. Cells were treated with the specified concentration of drug for 24 h. High speed live cell interferometry (HSLCI) was used to obtain single cell biomass measurements every ten minutes as described by Murray et al. (2018) [23]. Biomass measurements were aggregated to create plots depicting hourly cell growth rate.

### Single-cell RNA sequencing

Single-cell RNA sequencing (scRNAseq) was performed on four human basal-like TNBC cell lines- HCC-1143, HCC-1187, MDA-468 and SUM-149 and four human basal-like TNBC PDXs- HCI-001, WHIM2, WHIM30, and UCD52. ScRNAseq was performed using the Chromium Single Cell Gene Expression Kit (10X Genomics) per the manufacturer's protocol. Samples were aligned and gene expression calculated using the 10X Genomics CellRanger v3.1 software suite of tools, and dead/poor quality cell removal was done using an in-house R script utilizing the Seurat v3.1.5 package. PDX samples went through additional filtering and realignment to remove mouse cells prior to creating a final merged dataset containing only human cells using CellRanger. 10X Loupe Cell Browser v4.0.0 was used to visualize cell clusters and perform differential gene expression analyses across clusters [24,25]. ScRNAseq data is publicly available on the NCBI Gene Expression Omnibus (GEO Accession: GSE174391).

### Immunohistochemistry

Immunohistochemical staining was performed on formalin-fixed, paraffin-embedded tumors. Heat-induced antigen retrieval was performed in pH 9 Tris-EDTA using a Dakocytomatin Pascal Pressure Chamber. XPO1 (Cell Signaling Technology, 46249) antibody was diluted 1:200 in SignalStain Antibody Diluent (Cell Signaling Technology) and was applied to sections from the aforementioned tumors. Detection was performed using the Rabbit Dako EnVision System (Agilent K406511–2). Slides were imaged using Zeiss Axio Observer.

### Public dataset analyses

The PDX RNA-sequencing data was obtained from a previously published dataset (GEO Accession: GSE118942) [26]. Breast cancer TCGA gene expression data were obtained and analyzed using the curatedTCGAdata v.1.12.1 and TCGAutils v.1.10.1 R packages. The associated statistical analyses and visualizations were performed in R v.4.0.3 statistical environment. XPO1 expression was also assessed using a combined 855 breast cancer dataset [18] derived from four breast cancer microarray datasets (GSE2034, GSE12276, GSE2603, and NKI295) combined with reported clinical site(s) of first relapse [27]. In the original studies, all patient tissue samples were collected in accordance with IRB-approved protocols. Patients were grouped based on breast cancer intrinsic subtype. Patients with basal-like tumors (*N* = 140) were rank-ordered based on XPO1 expression and divided into quartiles. Kaplan-Meier curves were generated with GraphPad Prism V9.0.0.

### Basal-like TNBC PDXs

Four basal-like TNBC PDXs were used in this study: HCI-001, WHIM30, WHIM2, and UCD52. HCI-001 was obtained from the Huntsman Cancer Institute. WHIM2 and WHIM30 were obtained from Washington University, St. Louis. UCD52 was obtained from the University of Colorado. Tumor fragments were implanted in the mammary gland of female non-obese diabetic severe combined immunodeficient gamma (NSG) mice. Tumors were allowed to grow until they reached approximately 10 mm x 10 mm in size. Then, tumors were removed and prepped into a single-cell suspension according to the protocol described previously [17]. Single-cell suspensions of PDX cells were used for *in vitro* assays, HSLCI, or serial passaging into mice.

### *In vivo* drug treatment studies

All studies involving mice were in accordance with the VCU Institutional Animal Care and Use Committee (IACUC). Single cell suspensions were prepped from harvested PDX mammary gland tumors. Tumor cells were resuspended in Matrigel (Corning) and injected into the right mammary gland (250,000 cells per injection) of female non-obese diabetic severe combined immunodeficient gamma (NSG) mice. Drug treatment began when all tumors were ∼ 3 mm x 3 mm. Mice were randomized into treatment groups. All drugs were dissolved in a solution of 1% methylcellulose + 0.1% Tween-80. All drugs were administered via oral gavage. KPT-330 was administered thrice weekly at 5 mg/kg for 21 days. MLN9708 and GSK2126458 were administered twice weekly at the appropriate doses, 4 mg/kg and 2 mg/kg respectively, for 21 days. Mice receiving combination treatment received KPT-330 thrice weekly and the second drug twice weekly. Combination-treated mice never received both drugs on the same day. Tumor growth was monitored via biweekly caliper measurements. After 21 days, all mice were euthanized via CO_2_ asphyxiation followed by cervical dislocation. Tumors were then excised, weighed *ex vivo*, and photographed. Mice reaching maximum tumor burden prior to completion of the study were sacrificed, and their tumor measurements were not considered in endpoint analyses.

## Results

### High throughput drug screening of cell lines and PDXs identified cytotoxic drugs for basal-like TNBC

**Fig. 1:**
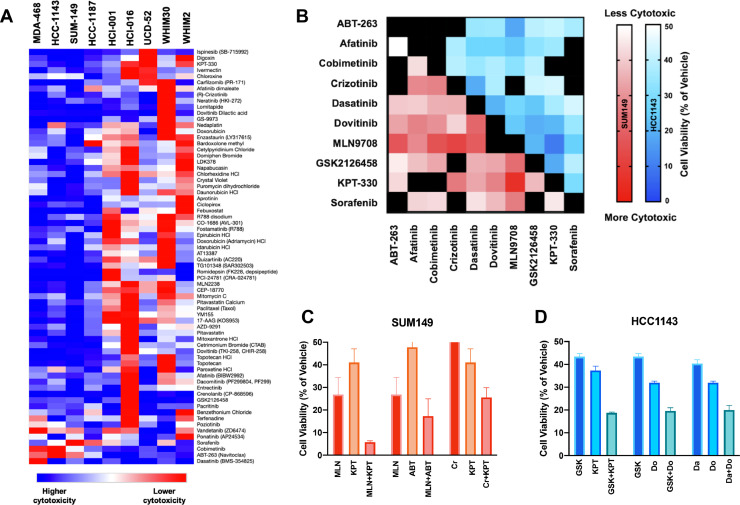
*High throughput screening (HTS) of 1363 drugs and preliminary assessment of drug combination cytotoxicity on human basal-like TNBC cell lines and PDXs***. (a)** TNBC cell lines were treated for 72 h with 10 µM of each drug. Luciferase-based imaging was used to assess viability relative to vehicle. The heatmap depicts relative cytotoxicity of 68 promising drugs of interest on human basal-like TNBC cell lines. Basal-like TNBC cell line HTS data was compared to basal-like PDX HTS data published previously [17]. **(b)** A cytotoxic dose of drug 1 (∼IC50) and drug 2 (∼IC50) was applied to HCC-1143 and SUM-149 cells in every possible two-drug combination. Heatmap depicts the relative cytotoxicity of each two-drug combination with darker colors depicting greater cytotoxicity. Black squares indicate combinations producing > 50% cell viability or combinations of the same drug. **(c, d)** Cytotoxicity of the two-drug combinations was compared to cytotoxicity of single agents. Three combinations that demonstrated significantly greater cytotoxicity or trended towards greater cytotoxicity than either single agent in HCC-1143 and/or SUM-149 were identified.

Since the majority (∼75%) of TNBCs are transcriptomically classified as basal-like [9], we utilized four basal-like human cell lines for these studies: HCC-1143, HCC-1187, MDA-468, and SUM-149. To best model aggressive, advanced disease, cell lines were chosen for their demonstrated ability to metastasize *in vivo* or relative chemoresistance *in vitro* [28,29,30]. The cytotoxic activity of 1363-drugs that have largely been FDA-approved for cancer/non-cancer indications was determined at a 10 µM dose; 68 therapeutic candidates that were strongly cytotoxic across the models were identified. These data were contrasted with cytotoxic responses of five human basal-like TNBC PDXs **(**Fig. 1**a)** [17]. Many of the drugs that were cytotoxic towards cell lines were also effective on PDX cell suspensions. Classes of effective drugs found to be previously well-tolerated in clinical trials were prioritized for further study. Ultimately, ten agents with unique molecular targets were selected for further evaluation in combinatorial studies (primary drug target in parentheses): ABT-263 (BCL-2), afatinib (EGFR), cobimetinib (MEK), crizotinib (ALK, ROS1), dasatinib (SRC), dovitinib (FGFR), MLN2238 (PSMB5), GSK2126458 (PI3K/mTOR), KPT-330 (XPO1), and sorafenib (BRAF). MLN9708, the citrate-bound version of MLN2238 which hydrolyzes to the biologically active form MLN2238 upon exposure to aqueous solutions or plasma, was used for *in vitro* and *in vivo* studies. Dose response curves were performed with the four human basal-like TNBC cell lines to identify the cytotoxicity profile of each agent **(Supplemental Fig. S1)**.

### Drugs of interest displayed increased cytotoxicity in combination

A single dose of each selected drug that killed approximately half of the cells compared to vehicle (IC50) was used in a combinatorial assay with every other drug at its pre-defined dose (**Supplemental Table S1)**. This led to the exploration of 45 unique drug combinations targeting distinct molecular pathways **(**Fig. 1**b)**. From this dataset, three drug combinations from each cell line that showed increased cytotoxicity in combination compared to as a single agent were identified **(**Fig. 1**c, d)**.

### Nuclear-export inhibitor-based combinations were synergistically cytotoxic in human basal-like TNBC cell lines

**Fig. 2:**
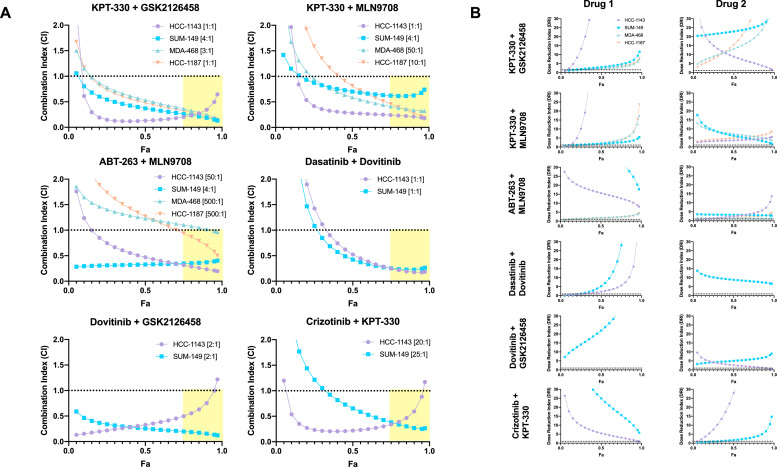
*Nuclear-export inhibitor-based combinations are synergistically cytotoxic in human basal-like TNBC cell lines***.** After 72 h of drug treatments, cell viability assessments were performed using CellTiter-Glo. Chou-Talalay drug combination analyses were performed to identify quantifiable synergistic cytotoxicity of pre-identified drug pairs on human basal-like TNBC cell lines. CI value > 1 indicates antagonism; CI value = 1 indicates additivity; CI value 〈< 1 indicates synergism. DRI 〉> 1 indicates a favorable reduction in drug dose when administered in combination at the ratio of drug 1: drug 2 indicated in brackets next to the cell line. Fa represents fraction inhibition, or fraction of cells killed. Fa is plotted on the x-axis against combination index **(a)** and dose reduction index **(b)**.

The Chou-Talalay Method of synergism analysis was utilized to identify quantifiable synergism with the following combinations: KPT-330 + GSK2126458, KPT-330 + MLN9708, ABT-263 + MLN9708, dasatinib + dovitinib, dovitinib + GSK2126458, and crizotinib + KPT-330) (Fig. 2) [20], [21], [22]. CompuSyn software was utilized to perform the Chou-Talalay Method of synergism analysis. CompuSyn software calculated combination index (CI) values and dose-reduction index (DRI) values for each combination. CI values > 1 indicate antagonism; CI value = 1 indicates additivity; CI values (<1 indicate synergism. The DRI value is a measure of fold reduction of drug dose when administered in combination as opposed to as a single agent; DRI ) >1 is favorable. Of the six combinations tested, two combinations demonstrated synergistic cytotoxicity at high levels of cell death across all four cell lines: KPT-330 + GSK2126458 and KPT-330 + MLN9708 **(**Fig. 2**)**.

### High speed live cell interferometry assessed cellular growth rate following drug treatment at the single-cell level

**Fig. 3:**
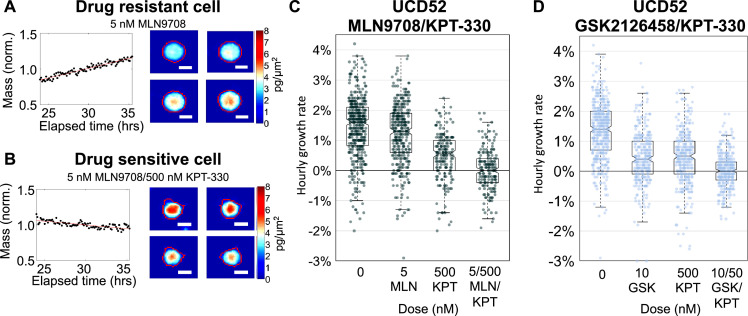
*Nuclear-export inhibitor-based combination treatments decrease hourly cell growth rate***.** A single-cell suspension of UCD52 tumor cells was plated and treated with the corresponding concentration of drug for 24 h. High speed live cell interferometry (HSLCI) was used to obtain single cell biomass measurements every eight minutes. Changes in cell biomass over time were analyzed to calculate an hourly growth rate. **(a)** Representative graph of a MLN9708 resistant cell growing at 2.7% +/- 0.09% over 24–36 h after drug treatment. Images depict one cell at four different time points. Scale bar: 10 μm. **(b)** Representative graph of a cell sensitive to the MLN9708/KPT-330 combination losing mass at −1.1% +/- 0.11% over 24–36 h after drug treatment. Images depict one cell at four different time points. Scale bar: 10 μm. **(c)** Combination treatment with KPT-330 and MLN9708 decreased median hourly growth rate to a greater extent than treatment with either single agent and reduced the population of growing single cells. Each dot on the box plot represents the measurement of a single cell as depicted in a and b. **(d)** Combination treatment with KPT-330 and GSK2126458 decreased median hourly growth rate to a greater extent than treatment with either single agent and reduced the population of growing single cells. *p*-values are listed in **Supplemental Table S2**.

Cell biomass measurements with high speed live cell interferometry (HSLCI) have been utilized to determine the effect of drugs on individual cells within a population, the proof-of-principle of which we have previously demonstrated with carboplatin and BRAF inhibitors [23,31]. In this approach, cell growth corresponds with increased cell mass **(**Fig. 3**a)**, and active cell death corresponds with decreasing mass **(**Fig. 3**b)**. In this study, UCD52 PDX cells were treated with vehicle, KPT-330, MLN9708, GSK2126458, KPT-330 + MLN9708, or KPT-330 + GSK2126458, respectively. Treatment with KPT-330, MLN9708, and GSK2126458 as single agents decreased hourly growth rate relative to vehicle. However, combination treatment with either KPT-330 and MLN9708 or KPT-330 and GSK2126458 decreased hourly growth rate to a significantly greater extent than treatment with a single agent alone **(**Fig. 3**c, d)**.

### XPO1 expression within basal-like TNBC samples

**Fig. 4:**
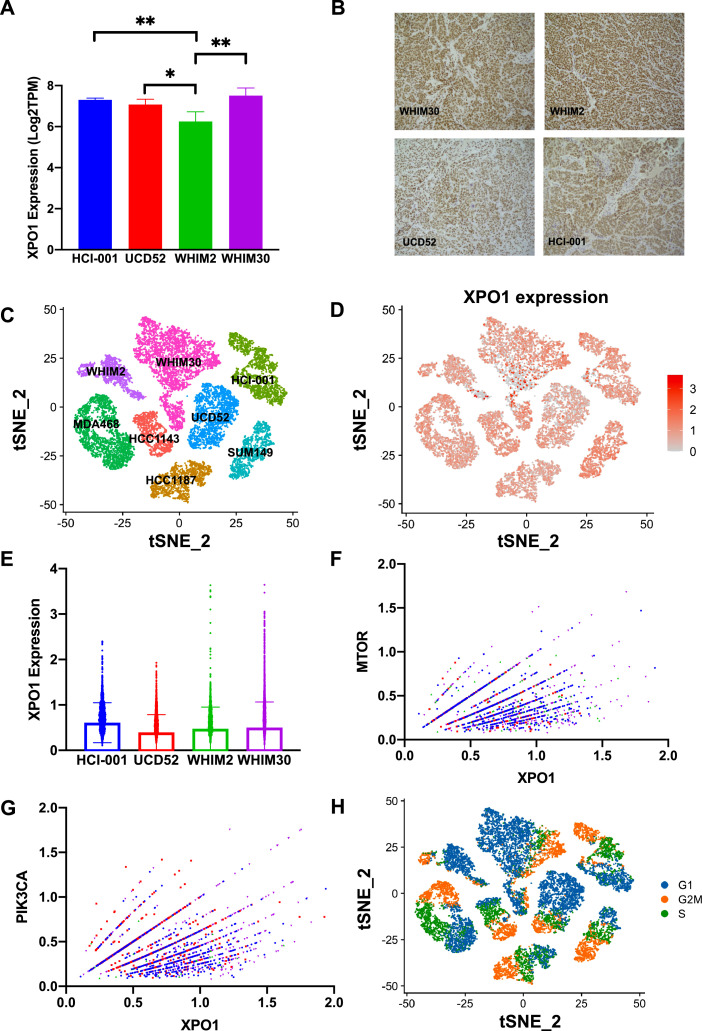
**XPO1 is heterogeneously expressed in human basal-like TNBC samples, and XPO1 expression is positively correlated with PIK3CA and MTOR expression at the single-cell level. (a)** Bulk RNA-sequencing of human basal-like TNBC PDX samples (HCI-001, UCD-52, WHIM2, and WHIM30) **(b)** Immunohistochemical staining on formalin-fixed, paraffin-embedded HCI-001, WHIM2, WHIM30, and UCD52 mammary gland tumors revealed positive XPO1 protein expression in all four PDX samples. Images were taken at 40X magnification. **(c, d)** Single cell RNA sequencing of human basal-like TNBC cell lines and PDXs **(e)** Box plot depicting single cell XPO1 expression values by PDX **(f, g)** Non-zero XPO1 expression values for single cells were plotted against non-zero single cell expression values for MTOR and PIK3CA (**h**) Cell cycle analysis (* *p* < 0.05, ** *p* < 0.01, *** *p* < 0.001). *p*-values are listed in **Supplemental Tables S3, S4, S5.**

Four basal-like patient-derived xenografts were utilized for a set of experiments *in vivo*, including HCI-001, UCD52, WHIM2, and WHIM30. Analysis of bulk RNA-sequencing (RNAseq) from these PDX samples revealed consistent, positive expression of XPO1 transcript across basal-like TNBC PDX samples **(**Fig. 4**a; Supplemental Table S3**), with WHIM2 showing significantly lower XPO1 expression than the other three PDXs. Immunohistochemistry found that XPO1 protein was expressed in all tested PDXs **(**Fig. 4**b)**. These data correlate with XPO1 immunohistochemistry performed by The Protein Atlas on 11 patient tumor samples where homogeneous moderate to high protein expression was found in > 75% of epithelial tumor cells for each sample. **(Supplemental Fig. S2)** [32], [33], [34]. ScRNAseq was performed on the 4 cell lines and the 4 PDXs used in this study **(**Fig. 4**c)**. ScRNAseq revealed that XPO1 was abundantly expressed in the majority of the basal-like cells, with more heterogenous expression present within the PDX samples than the cell lines **(**Fig. 4**d)**. In each PDX, a subset of cells demonstrated XPO1 overexpression compared to the bulk of the population (Fig. 4**e**). At the cellular level, there were significant positive correlations between XPO1 expression and MTOR expression as well as XPO1 expression and PIK3CA expression (Fig. 4**f, g; Supplemental Tables S4, S5**). Interestingly, there was also a significant positive correlation between expression of XPO1 and expression of MKI67, a known marker of proliferation (**Supplemental Fig. S3; Supplemental Table S6)** [35]. Pearson correlation values were determined for XPO1 and each gene within the PAM50 gene signature. Interestingly, XPO1 was positively correlated with all 11-proliferation associated genes (**Supplemental Fig. S4**) [35]. Correspondingly, cell cycle analysis revealed that a large proportion of cells that highly expressed XPO1 also expressed G2M genes (Fig. 4**h**). High G2M pathway scores have been associated with high expression of other proliferation-related gene sets and worse clinico-pathologic features [36].

### KPT-330 and GSK2126458 combination treatment demonstrates antitumor activity in basal-like TNBC PDXs

**Fig. 5:**
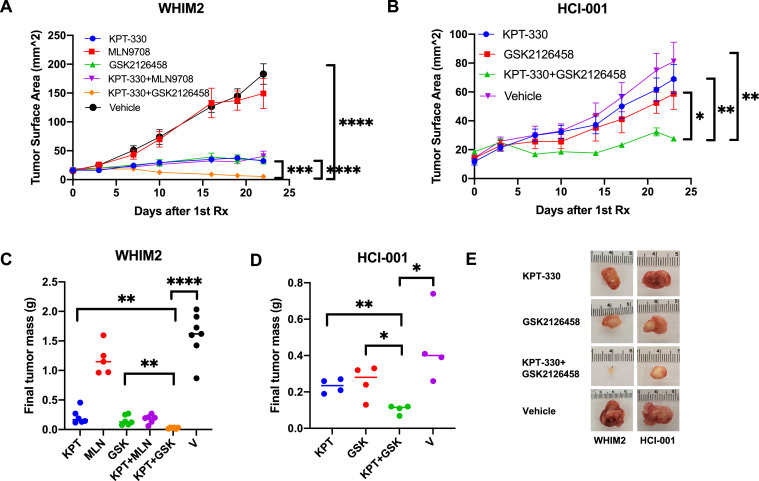
*KPT-330 and GSK2126458 demonstrate antitumor activity in vivo.* Mice bearing **(a)** WHIM2 or **(b)** HCI-001 mammary gland tumors were randomized and treated with drugs once tumors were ∼ 3 mm x 3 mm. Mice received vehicle orally (PO) thrice weekly, 5 mg/kg KPT-330 PO thrice weekly, 4 mg/kg MLN9708 PO twice weekly, 2 mg/kg GSK2126458 PO twice weekly, KPT-330 and MLN9708 regimens, or KPT-330 and GSK2126458 regimens. Tumor growth was graphed by obtaining biweekly tumor caliper measurements. Tumor surface area was defined as length x width. **(c, d)** At experimental endpoint, tumors were removed from mice and weighed *ex vivo* to determine final tumor mass. **(e)** Representative images of extracted treated tumors are shown. Error bars represent SEM (* *p* < 0.05, ** *p* < 0.01, *** *p* < 0.001, **** *p* < 0.00001). *p*-values are listed in **Supplemental Tables S7, S8**.

The efficacy of KPT-330, MLN9708, GSK2126458, or combination treatment with KPT-330 and MLN9708 or GSK2126458 for inhibiting tumor growth *in vivo* was evaluated on four basal-like TNBC PDXs: HCI-001, UCD52, WHIM2, and WHIM30 (Fig. 5**; Supplemental Figs. S5, S6**). The patients from whom the HCI-001 and WHIM2 PDXs were derived presented with distant metastases and subsequently died from metastatic disease; the patient from whom the WHIM30 PDX was derived presented with no metastases [37,38]. HCI-001, WHIM2, and WHIM30 cells were isolated from the patient prior to the initiation of treatment [37,39]. There is no clinical data available for UCD52. Alzubi et al. (2019) and Turner et al. (2018) demonstrated that each of these PDXs had metastatic potential [26,40]. Initial *in vivo* treatments found that the combination of KPT-330 and GSK2126458, but not KPT-330 and MLN9708, resulted in significantly smaller tumors than treatment with either monotherapy in the WHIM2 PDX **(Supplemental Fig. S5).** Further studies found that combination treatment with KPT-330 and GSK2126458 produced antitumor activity in all four PDXs **(Supplemental Fig. S6**). In WHIM2 and HCI-001, the combination of KPT-330 and GSK2126458 demonstrated significantly greater antitumor activity than either single agent, as determined by final tumor surface area and tumor mass **(**Fig. 5**)**. Mice did not demonstrate any signs of acute toxicity, and on average, there were negligible changes in mouse mass (± 5%). Hematological analysis of drug treated mice did not find any appreciable differences in erythrocytes or thrombocytes between vehicle and drug combination treated mice.

### XPO1 expression across intrinsic subtypes and association with metastasis-free survival

**Fig. 6:**
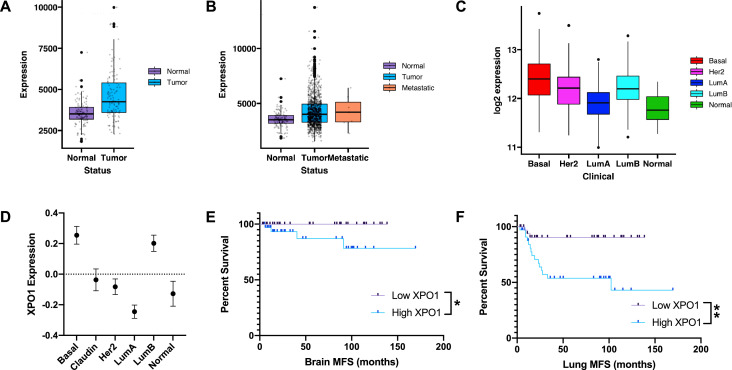
*XPO1 is a relevant clinical biomarker in basal-like breast cancer.* Public datasets were used to quantify XPO1 expression and relationship with outcome. **(a)** The Cancer Genome Atlas (TCGA) RNA-sequencing dataset was used to quantify XPO1 expression in patient-matched normal-tumor paired samples. **(b)** Box plots of TCGA normal breast, breast tumors, and metastases **(c)** TCGA breast tumor data for XPO1 expression across intrinsic subtypes. **(d)** A separate 855-patient RNA-sequencing dataset was also used to evaluate varying levels of XPO1 expression across breast cancer subtypes. **(e, f)** Basal-like patients (*N* = 140) were divided into quartiles based on XPO1 expression, and Kaplan-Meier analyses were performed on the top/bottom quartiles for brain and lung metastasis free survival (MFS), respectively. (* *p* < 0.05, ** *p* < 0.01, *** *p* < 0.001). *p*-values are listed in **Supplemental Tables S9, S10**.

The Cancer Genome Atlas (TCGA) data for ductal breast carcinoma was used to evaluate XPO1 expression across different normal and cancerous breast sample groups. In patient-matched normal breast and breast tumor samples, XPO1 was significantly overexpressed (*p* < 0.001) in tumors as compared to normal tissue **(**Fig. 6**a)**. Across all TCGA samples, XPO1 was, on average, significantly overexpressed in TCGA breast cancer patient samples compared to adjacent normal tissue **(**Fig. 6**b)**. When XPO1 expression was compared across TCGA breast cancer samples, basal-like tumors showed significantly greater XPO1 expression than other breast cancer subtypes **(**Fig. 6**c; Supplemental Table S9).** Similar trends in XPO1 expression were observed utilizing a separate public dataset **(**Fig. 6**d; Supplemental Table S10)** [18]. Interestingly, basal-like tumors with the highest XPO1 expression were correlated with worse metastasis-free survival **(**Fig. 6**e, f)**.

## Discussion

In these studies, we sought to identify novel therapeutic combinations that are synergistically cytotoxic towards basal-like breast cancers. Initial screening studies supported targeting XPO1 with KPT-330 (trade name selinexor), which is an FDA-approved therapeutic for multiple myeloma and lymphoma. Arango et al. (2017) demonstrated the promising preclinical antitumor activity of KPT-330 against TNBC [41]. Soon after, KPT-330 was explored in a metastatic TNBC Phase II clinical trial [42]. While KPT-330 was well-tolerated, it did not produce objective responses. The clinical benefit rate, however, was 30%. This is similar to the 25% response rate we observed herein as a single-agent. The investigators suggested that future studies of KPT-330 in TNBC focus on a combinatorial or biomarker-driven approach [42].

KPT-330 has previously been shown to synergize with a variety of chemotherapies and targeted drugs in both TNBC and other models of cancer. Arango et al. (2017) demonstrated that KPT-330 synergized with select chemotherapies in TNBC PDXs *in vivo* [41]. There is also abundant preclinical and clinical evidence for synergism between KPT-330 and proteasome inhibitors in other cancers, including myeloma and high-grade glioma [43], [44], [45]. Studies have found that both selinexor and proteasome inhibitors inhibit the NF-kB pathway, and in this way, combinatorial therapy may induce synergistic cytotoxicity via dual inhibition of the NF-kB pathway [44,45]. One previous study demonstrated synergism of KPT-330, an mTOR inhibitor (everolimus), and dexamethasone in non-Hodgkin's lymphoma [46]. To our knowledge, however, KPT-330 has not been shown to synergize with a dual PI3K/mTOR inhibitor in any model of cancer. Thus, dual inhibition of XPO1 and PI3K/mTOR represents a novel molecular interaction and means of producing antitumor activity. GSK2126458 (trade name omipalisib) was evaluated in combination with trametinib in a phase Ib dose-escalation study for solid tumors [47] but yielded minimal evaluable responses, perhaps due to overlapping toxicities that prevented exposure to a sufficient drug dose. In another phase I advanced solid tumor clinical trial, treatment with single agent GSK2126458 was well-tolerated and produced durable objective responses in patients with several tumor types, including breast cancer [48]. Future studies that aim to investigate this combination, or other combinations targeting PI3K/mTOR, should likely focus on scheduling of the drug combinations to minimize potential toxicity.

We found that within basal-like patients, XPO1 overexpression was positively correlated with brain relapse and lung relapse. High cellular proliferation rates have long been known to be drivers of metastatic ability [49]. Given our findings that the cells with the highest XPO1 expression also have high expression of markers of proliferation, we propose that targeting XPO1 in combination with other compensatory pathways will provide benefit for surgically inaccessible metastases. Co-targeting of XPO1 and PI3K/mTOR could also serve as an alternative therapy for chemotherapy-resistant tumors, such as the carboplatin-insensitive WHIM2 PDX [40]. Analysis of scRNAseq data revealed that XPO1 and PIK3CA/MTOR expression were positively related at the single cell level. Furthermore, combination treatment with KPT-330 and GSK2126458 produced antitumor activity in all four PDXs. Given the promising preliminary data, further preclinical study of KPT-330 and GSK2126458 in the TNBC setting is warranted, especially for patients who are no longer responding to standard of care chemotherapeutics. This novel combination may have the potential to impact patient treatment decisions and improve patient outcomes.

## Data availability statement

All data are available upon publication. The scRNAseq data is available at the NCBI Gene Expression Omnibus: GSE174391.

## CRediT authorship contribution statement

**Narmeen S. Rashid:** Conceptualization, Methodology, Formal analysis, Investigation, Writing – original draft, Visualization. **Nicole S. Hairr:** Investigation, Writing – review & editing. **Graeme Murray:** Methodology, Formal analysis, Investigation, Writing – review & editing. **Amy L. Olex:** Software, Formal analysis, Data curation, Writing – review & editing. **Tess J. Leftwich:** Investigation, Writing – review & editing. **Jacqueline M. Grible:** Investigation, Writing – review & editing. **Jason Reed:** Resources, Supervision, Writing – review & editing. **Mikhail G. Dozmorov:** Software, Formal analysis, Writing – review & editing, Visualization, Supervision. **J. Chuck Harrell:** Conceptualization, Methodology, Resources, Writing – review & editing, Supervision, Project administration, Funding acquisition.

## Declaration of Competing Interest

The authors declare that they have no known competing financial interests or personal relationships that could have appeared to influence the work reported in this paper.

## Appendix Supplementary materials

Image, application 1

Image, application 2

Image, application 3
