# Supplementary material for: Identification of nuclear export inhibitor-based combination therapies in preclinical models of triple-negative breast cancer
Source: Transl Oncol. 2021 Oct 7;14(12):101235. doi: 10.1016/j.tranon.2021.101235 (PMC8512760; doi:10.1016/j.tranon.2021.101235)
Supplement: Supplementary file 1 [file mmc1.docx]

**Supplementary Information**

**Supplemental Table S1:** Drug doses used in combination assay shown in Figure 1.

| **Drug** | **HCC-1143 Dose (μM)** | **SUM-149 Dose (μM)** |
| --- | --- | --- |
| ABT-263 | 10.00 | 0.100 |
| Afatinib | 4.000 | 0.500 |
| Cobimetinib | 10.00 | 5.000 |
| Crizotinib | 3.000 | 3.000 |
| Dasatinib | 0.300 | 0.100 |
| Dovitinib | 0.500 | 0.500 |
| Ixazomib | 0.050 | 0.200 |
| Omipalisib | 0.100 | 10.00 |
| Selinexor | 0.050 | 0.300 |
| Sorafenib | 10.00 | 10.00 |

**Supplemental Table S2:** P-values for HSCLI experiments on a single cell suspension of UCD52 PDX cells. Significant values (p<.05) are bolded.

**Combination 1: KPT-330 + MLN9708**

|  | Vehicle | KPT-330 | MLN9708 | Combination |
| --- | --- | --- | --- | --- |
| Vehicle | - | - | - | - |
| KPT-330 | **1.10E-27** | - | - | - |
| MLN9708 | **.0038** | **8.90E-17** | - | - |
| Combination | **2.70E-53** | **6.12E-08** | **8.29E-38** | - |

**Combination 2: KPT-330 + GSK2126458**

|  | Vehicle | KPT-330 | GSK2126458 | Combination |
| --- | --- | --- | --- | --- |
| Vehicle | - | - | - | - |
| KPT-330 | **1.01E-33** | - | - | - |
| GSK2126458 | **9.21E-34** | 0.9632 | - | - |
| Combination | **1.56E-77** | **3.36E-11** | **1.71E-11** | - |

**Supplemental Table S3:** P-values for XPO1 expression in four basal-like TNBC PDXs as shown in Figure 4a. One-way ANOVA followed by post-hoc Tukey’s HSD (Honest Significant Difference) test was used to detect significant differences. Significant values (p<.05) are bolded.

| **PDX comparison** | **P-value** |
| --- | --- |
| HCI-001 vs. UCD52 | 0.7416 |
| HCI-001 vs. WHIM2 | **0.0076** |
| HCI-001 vs. WHIM30 | 0.8305 |
| UCD52 vs. WHIM2 | **0.0150** |
| UCD52 vs. WHIM30 | 0.2015 |
| WHIM2 vs. WHIM30 | **0.0011** |

**Supplemental Table S4:** Pearson’s correlation coefficients were calculated to assess the strength and relationship between XPO1 expression and MTOR expression in four basal-like TNBC PDXs. Significant values (p<.05) are bolded.

| **PDX** | **Sample Size (# of cells)** | **r** | **P-value** |
| --- | --- | --- | --- |
| HCI-001 | 614 | 0.2333 | **<0.0001** |
| UCD52 | 215 | 0.1633 | **0.0165** |
| WHIM2 | 303 | 0.2590 | **<0.0001** |
| WHIM30 | 550 | 0.3733 | **<0.0001** |

**Supplemental Table S5:** Pearson’s correlation coefficients were calculated to assess the strength and relationship between XPO1 expression and PIK3CA expression in four basal-like TNBC PDXs. Significant values (p<.05) are bolded.

| **PDX** | **Sample Size (# of cells)** | **r** | **P-value** |
| --- | --- | --- | --- |
| HCI-001 | 603 | 0.2557 | **<0.0001** |
| UCD52 | 481 | 0.2448 | **<0.0001** |
| WHIM2 | 107 | 0.1467 | 0.1316 |
| WHIM30 | 706 | 0.4752 | **<0.0001** |

**Supplemental Table S6:** Pearson’s correlation coefficients were calculated to assess the strength and relationship between XPO1 expression and MKI67 expression in four basal-like TNBC PDXs. Significant values (p<.05) are bolded.

| **PDX** | **Sample Size (# of cells)** | **r** | **P-value** |
| --- | --- | --- | --- |
| HCI-001 | 1131 | 0.2591 | **<0.0001** |
| UCD52 | 689 | 0.2450 | **<0.0001** |
| WHIM2 | 392 | 0.4209 | **<0.0001** |
| WHIM30 | 1241 | 0.3785 | **<0.0001** |

**Supplemental Table S7:** P-values for WHIM2 *in vivo* drug experiments shown in Figure 5a, c. *t*-tests were performed to compare tumor surface areas between all treatment conditions at the beginning of treatment and at the endpoint. *t*-tests were also performed to compare final tumor mass between all treatment conditions. Significant values (p<.05) are bolded.

| **Treatment group comparison** | **Tumor Growth**  **Day 1 Day 22** | | **Tumor Masses** |
| --- | --- | --- | --- |
| Untreated vs. KPT-330 | 0.6858 | **<0.0001** | **<0.0001** |
| Untreated vs. GSK2126458 | 0.3943 | **<0.0001** | **<0.0001** |
| Untreated vs. KPT+GSK | 0.7860 | **<0.0001** | **<0.0001** |
| KPT-330 vs. GSK2126458 | 0.5164 | 0.9002 | 0.3593 |
| KPT-330 vs. KPT+GSK | 0.8477 | **<0.0001** | **0.0045** |
| GSK2126458 vs. KPT+GSK | 0.4316 | **0.0007** | **0.0033** |

**Supplemental Table S8:** P-values for HCI-001 *in vivo* drug experiment shown in Figure 5b, d. *t*-tests were performed to compare tumor surface areas between all treatment conditions at the beginning of treatment and at the endpoint. *t*-tests were also performed to compare final tumor mass between all treatment conditions. Significant values (p<.05) are bolded.

| **Treatment group comparison** | **Tumor Growth**  **Day 1 Day 23** | | **Tumor Masses** |
| --- | --- | --- | --- |
| Untreated vs. KPT-330 | 0.3821 | 0.4966 | 0.0815 |
| Untreated vs. GSK2126458 | 0.9205 | 0.2403 | 0.1329 |
| Untreated vs. KPT+GSK | 0.1240 | **0.0073** | **0.0153** |
| KPT-330 vs. GSK2126458 | 0.3575 | 0.5112 | 0.6694 |
| KPT-330 vs. KPT+GSK | 0.0546 | **0.0064** | **0.0014** |
| GSK2126458 vs. KPT+GSK | 0.0597 | **0.0287** | **0.0200** |

**Supplemental Table S9:** P-values for XPO1 expression in different BRCA subtypes as shown in Figure 6c. One-way ANOVA followed by post-hoc Tukey’s HSD (Honest Significant Difference) test was used to detect significant differences. Significant values (p<.05) are bolded.

| **Subtype comparison** | **P-value** |
| --- | --- |
| Basal vs. HER2 | **0.0007** |
| Basal vs. LumA | **<0.0001** |
| Basal vs. LumB | **0.0004** |
| Basal vs. Normal | **<0.0001** |

**Supplemental Table S10:** P-values for XPO1 expression in different BRCA subtypes as shown in Figure 6d. A one-way ANOVA with multiple comparisons was performed to determine whether XPO1 expression is significantly higher in basal-like tumors compared to other BRCA subtypes. Significant values (p<.05) are bolded.

| **Subtype comparison** | **P-value** |
| --- | --- |
| Basal vs. Claudin | **0.0169** |
| Basal vs. HER2 | **0.0004** |
| Basal vs. LumA | **<0.0001** |
| Basal vs. LumB | 0.9843 |
| Basal vs. Normal | **0.0010** |

**Supplemental Figure S1.** **Dose responses of human basal-like cell lines to drugs of interest**. Graphs of cell viability (percent of vehicle) generated from luciferase-based imaging following treatment with increasing doses of the indicated drugs. Graphs only display cytotoxic doses, or doses that produced < 100% cell viability. Two independent experiments were performed in triplicate and averaged. Error bars depict ± SD.


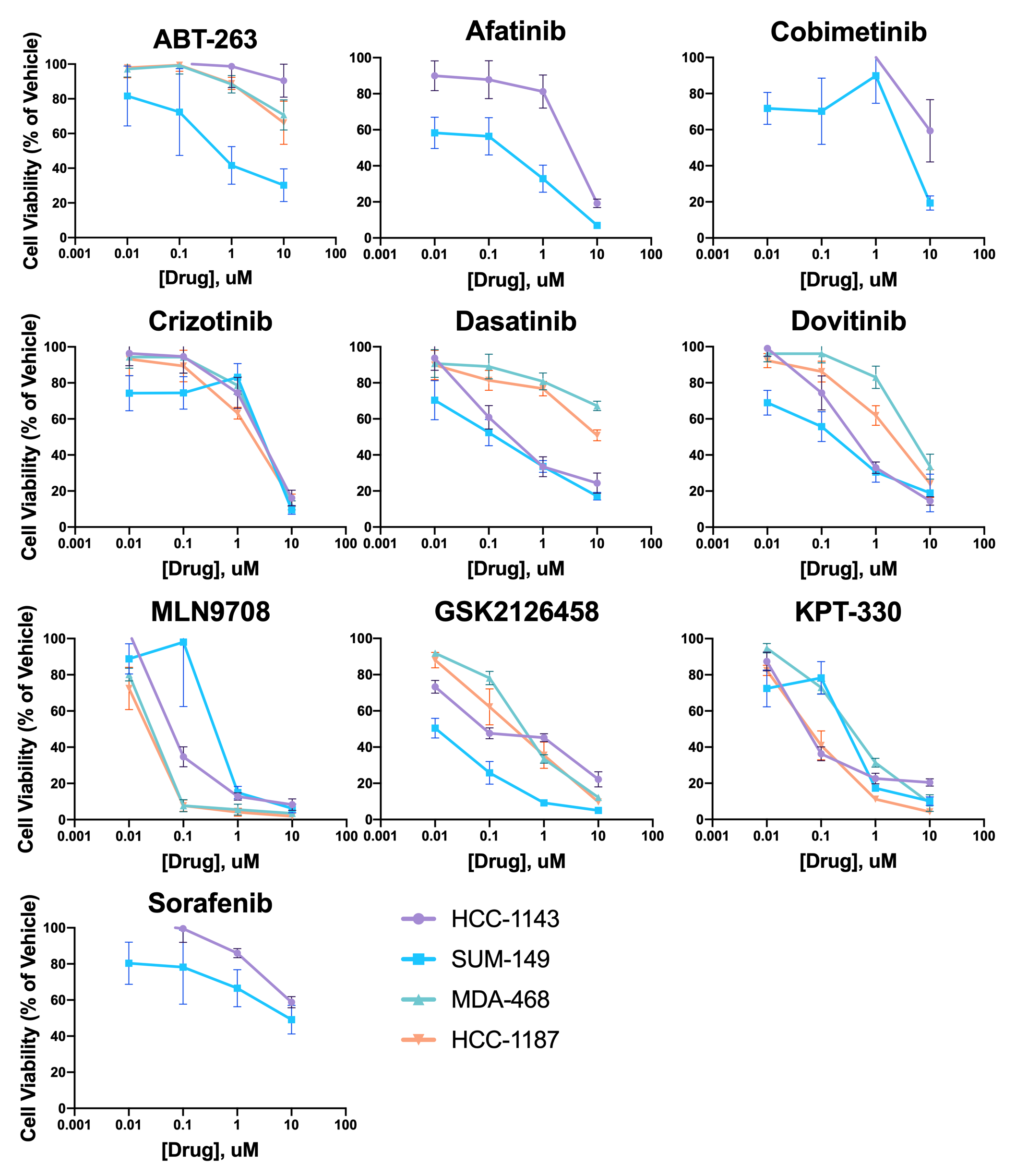


**Supplemental Figure S2.** Immunohistochemical staining of primary breast carcinomas reveals moderate to strong intensity staining for XPO1. This indicates positive, abundant XPO1 protein expression in primary breast tumors. Images available from the Human Protein Atlas ([v20.proteinatlas.org/ENSG00000082898-XPO1/pathology/breast+cancer](https://urldefense.com/v3/__http:/v20.proteinatlas.org/ENSG00000082898-XPO1/pathology/breast*cancer__;Kw!!JqxBPMk!0ARLVIc2ZJ9CPVCAPqREU9LfCQV2xoSSx4ujjgcochwGAxqwGvWwGJtDINePyJ8Fuw$)).


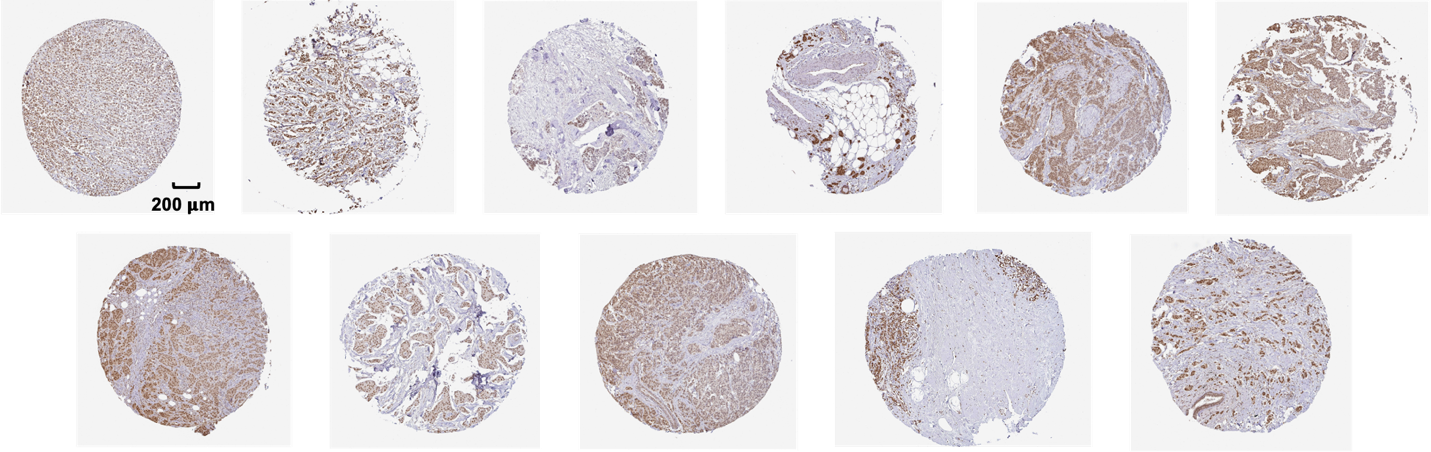


**Supplemental Figure S3.** Non-zero XPO1 expression values for single cells were plotted against non-zero single cell expression values for MKI67, revealing a significant positive correlation between XPO1 expression and MKI67 expression at the single cell level.


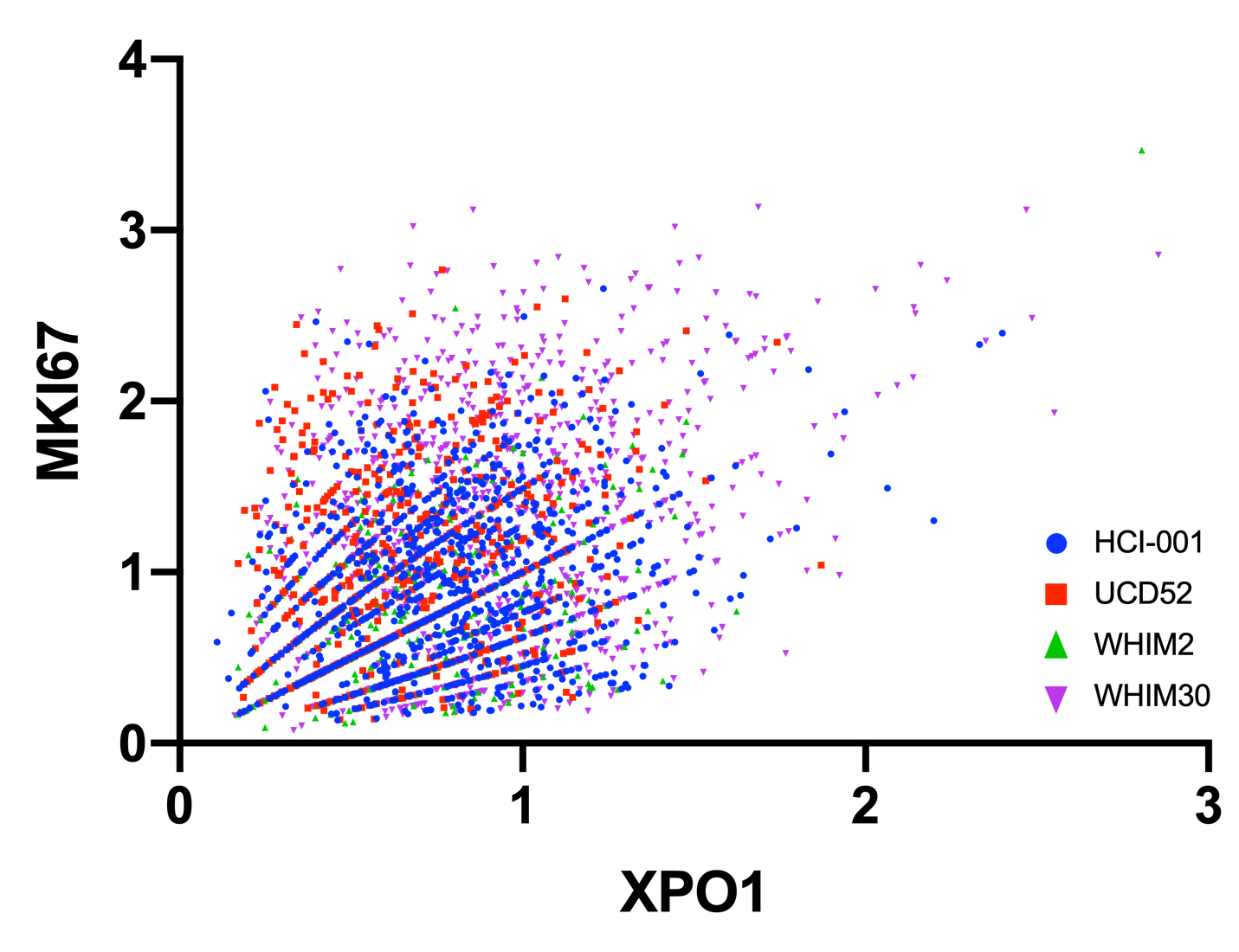


**Supplemental Figure S4.** Heatmap of an all-by-all Pearson's correlation coefficient analysis using normalized gene expression profiles for the PAM50 gene set across all 16,775 cells in the data set. Positive values (red) indicate that the expression of the two genes is positively correlated. Negative values (blue) indicate that the expression of the two genes is negatively correlated.

* denote genes associated with proliferation (Martín et al., 2013)

**Supplemental Figure S5.** WHIM2 *in vivo* drug studies represented as independent experiments (*N* = 17, *N* = 30). * In the second experiment, the two mice with the largest and smallest tumors from each treatment cohort (with the exception of MLN9708-treated tumors which reached burden prior to the end of the study) were kept alive to assess long-term survival following treatment.


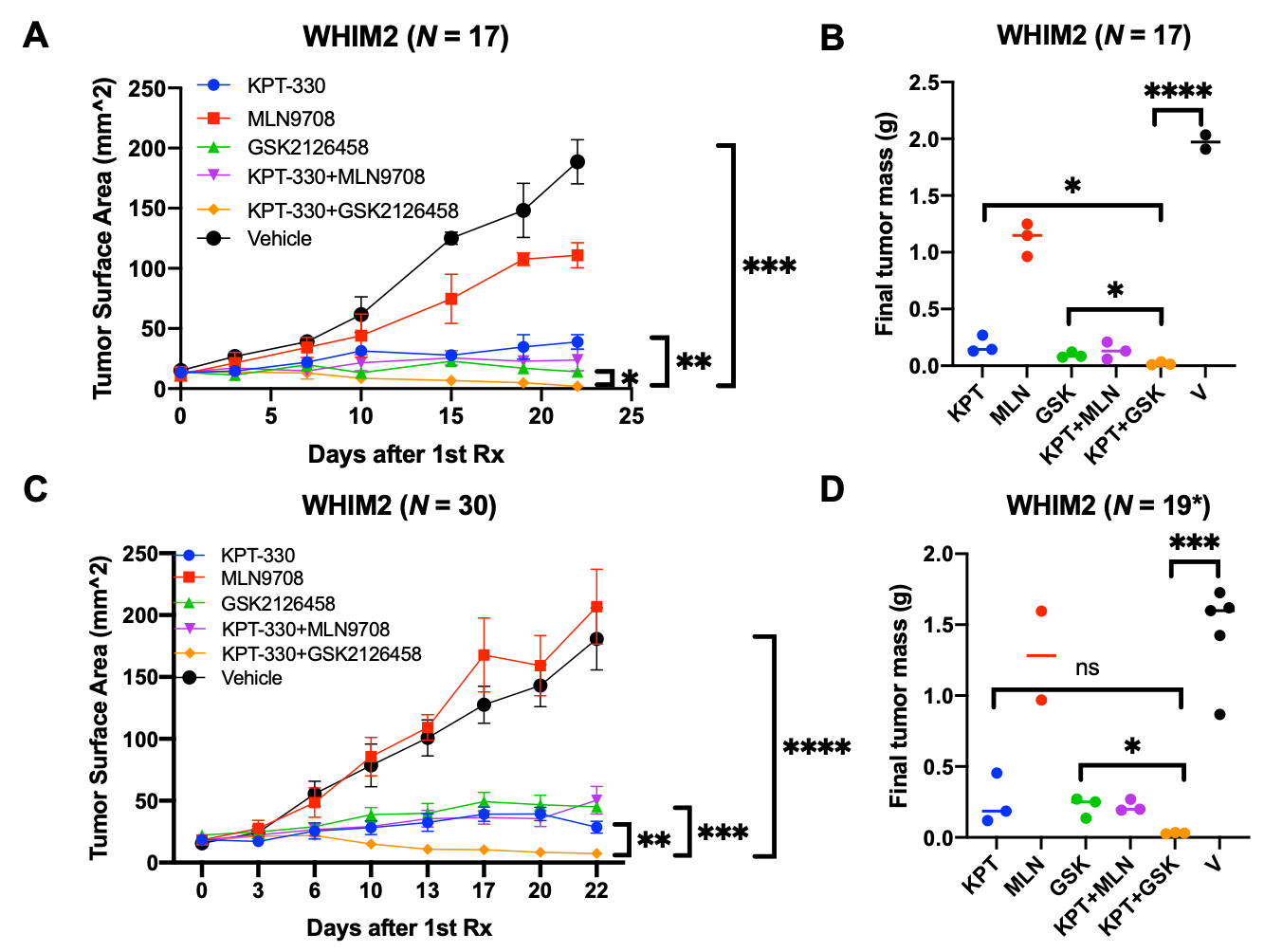


**Supplemental Figure S6.** *In vivo* drug studies were also performed on mice injected with UCD52 (*N* = 24) and WHIM30 (*N* = 16) mammary gland tumors in parallel to WHIM2 and HCI-001. Oral gavage appeared to inhibit UCD52 tumor growth, so treatment was stopped after 17 days, and vehicle tumors were allowed to grow to burden. Mice with WHIM30 tumors were treated for 28 days instead of 21 days in order to allow for vehicle tumors to reach burden. Drugs of interest demonstrated no significant antitumor activity as single agents on UCD52 or WHIM30 tumors. There is trending increased antitumor activity with combination therapy.


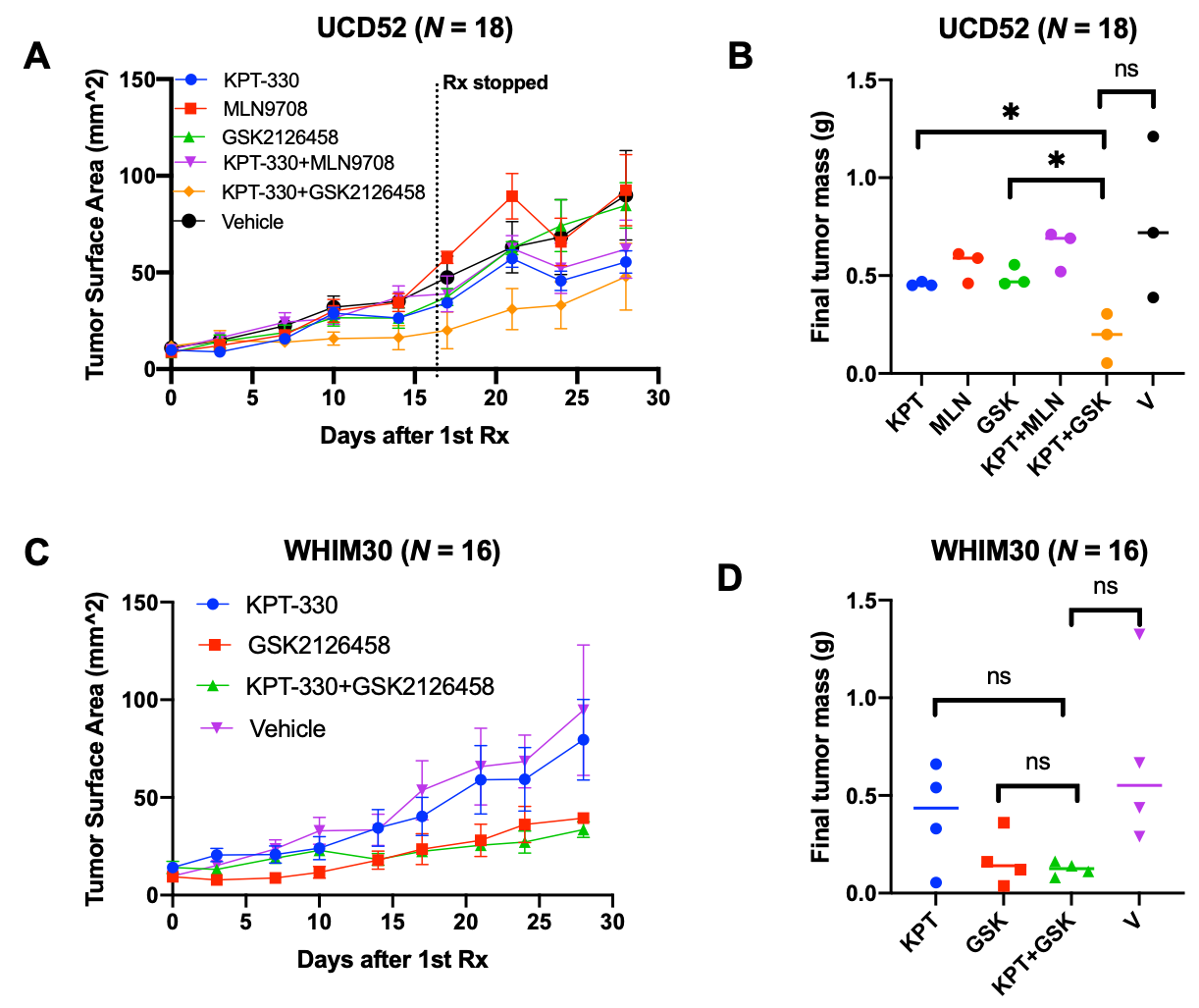


**Expanded Methods: scRNASeq Processing and Quality Control**

**Single-Cell RNA Sequencing**

Single-cell RNA sequencing was performed on four human basal-like TNBC cell lines- MDA468, HCC1143, HCC1187, and SUM149- and four human basal-like TNBC PDXs- HCI-001, WHIM2, WHIM30, and UCD52. Cell lines were harvested and re-suspended in PBS. PDXs were prepped into a single-cell suspension and re-suspended in HF buffer. Cell viability and count was assessed using the Countess 3 Automatic Cell Counter (Thermo Fisher Scientific). Single-cell RNA sequencing was performed using the Chromium Single Cell Gene Expression Kit (10X Genomics) and the provided protocol.

**Initial QC**

FastQC v0.11.8 (Andrews 2018) and MultiQC v1.7 (Ewels et al. 2016) were used for assessing read quality of the demultiplexed FastQ files received from the sequencing facility.

**Alignment and Barcode Filtering**

Samples were aligned and merged using the 10X Genomics CellRanger v3.1 software suite of tools, and dead/poor quality cell removal was done using an in-house R script utilizing the Seurat v3.1.5 package. To remove mouse cells, the PDX samples were additionally run through multiple rounds of alignment, barcode filtering, and conversion back to fastq format with CellRanger. Details of this process and a figure summarizing the workflow for PDX and TNBC samples is provided below.

*Step 1 -Initial alignment and human cell extraction:* Initial alignment was done using the 10X Genomics merged human and mouse genome (hg19mm10) for the PDX samples and the human genome (grch38) for the TNBC samples with the CellRanger “count” algorithm and the expected number of cells set to 5000. At this point, TNBC samples are moved to Step 2 while PDX samples undergo additional processing. Briefly, the resulting GEM file was used to identify the barcodes of all cells with the majority of reads aligning to the human genome only, thus filtering out poor quality cells, mouse cells, and multiplet cells for each sample as determined by the default filters of CellRanger. This list of barcodes was input into the CellRanger “subset-bam” algorithm in order to filter the aligned PDX BAM files to only those reads assigned to a valid human barcode. The filtered BAM file was then converted to a fastq file using the CellRanger script “bamtofastq”. These filtered fastq files were then re-aligned to the human (grch38) genome by CellRanger with the Force Cells option (instead of expected cells) being set to the same number of cells identified by the GEM file for each sample to turn off the default background filtering in the “count” algorithm (per correspondence with 10X support team). The resulting aligned data for the PDX samples was then moved to Step 2.

*Step 2 - Remove poor quality human cells:* We developed and ran an in-house R script using the Seurat package over the gene expression data from the human cells in all samples to identify those that may be dying, empty droplets, or multiplets. Thresholds for removing poor quality cells are calculated for each sample individually using the median absolute deviation (MAD). Dying cells are identified by assessing the percent of total mitochondrial gene expression in each sample. Any cell with a mitochondrial expression percent of more than 3 MADs above the median percent across all cells with < 50% mitochondrial expression in the sample were removed. Potential empty droplets or additional dying cells were identified by removing all cells where the number of genes identified (nFeature in Seurat) was less than 3 MADs below the median across all cells. Similarly, potential multiplets were identified by removing all cells that had a nFeature greater than 3 MADs above the median across all cells, or had an nCount (the number of unique molecules detected in a cell) greater than 3 MADs above the median across all cells. Output consisted of a list of barcodes for cells to keep and a list of barcodes for cells to remove. After filtering poor quality human cells, the list of barcodes to keep was input into the CellRanger “subset-bam” algorithm in order to filter the aligned BAM files for each sample followed by conversion to fastq files using the CellRanger script “bamtofastq”.

*Step 3 - Generate final filtered data set:* The fastq files generated from Step 2 were re-aligned to the GRCh38 reference genome using CellRanger with the Force Cells option set to the final number of cells remaining after filtering. The resulting output files from this alignment are those used in merging and other down-stream analyses.

**Sample Merging, Cell Clustering, and Gene Expression Analysis**

Once each sample had been aligned and filtered, the resulting feature matrix file from each was input into the CellRanger “aggr” algorithm to merge all three samples into a single CellRanger report formatted as a Loupe file. Cell clustering was done by the “aggr” pipeline using default settings. We used the 10X Loupe Cell Browser tool to visualize cell clusters and perform differential gene expression analyses across clusters.

Andrews, Simon. 2018. Babraham Bioinformatics - FastQC A Quality Control Tool for High Throughput Sequence Data. Babraham.

Ewels, Philip, Måns Magnusson, Sverker Lundin, and Max Käller. 2016. “MultiQC: Summarize Analysis Results for Multiple Tools and Samples in a Single Report.” Bioinformatics 32(19):3047–48.
